# An unusual case of *Erysipelothrix rhusiopathiae* prosthetic joint infection from the Canadian Arctic: whole genome sequencing unable to identify a zoonotic source

**DOI:** 10.1186/s12879-019-3913-7

**Published:** 2019-03-25

**Authors:** Michael Groeschel, Taya Forde, Shannon Turvey, A. Mark Joffe, Catherine Hui, Prenilla Naidu, Fabien Mavrot, Susan Kutz, Ameeta E. Singh

**Affiliations:** 1 Department of Medical Microbiology and Immunology, Faculty of Medicine and Dentistry, University of Alberta, Edmonton, Alberta Canada; 2 Institute of Biodiversity, Animal Health and Comparative Medicine, University of Glasgow, Glasgow, UK; 3 Division of Infectious Diseases, Department of Internal Medicine, Faculty of Medicine and Dentistry, University of Alberta, 3B20-11111 Jasper Avenue, Edmonton, AB T5K 0L4 Canada; 4 Division of Orthopaedic Surgery, Department of Surgery, Faculty of Medicine and Dentistry, University of Alberta, Edmonton, Alberta Canada; 5 Provincial Laboratory for Public Health, Edmonton, Alberta Canada; 6 Department of Ecosystem and Public Health, Faculty of Veterinary Medicine, University of Calgary, Calgary, Alberta Canada

**Keywords:** *Erysipelothrix rhusiopathiae*, Prosthetic joint infection, Septic arthritis, Whole genome sequencing

## Abstract

**Background:**

*Erysipelothrix rhusiopathiae* is a zoonotic pathogen that causes erysipeloid and is most frequently associated with exposure to domestic swine. Infection of native and prosthetic joints is a rarely reported manifestation.

**Case presentation:**

We describe a case of *E. rhusiopathiae* prosthetic joint infection in a woman with a history of exposure to wild animals in the Canadian Arctic. Patient management involved a 1-stage surgical revision exchange with an antibiotic impregnated cement spacer and 6 weeks of intravenous penicillin G followed by 6 weeks of oral amoxicillin. Ten previously reported cases of *E. rhusiopathiae* joint infection are reviewed. Recent increases in mortality due to infection with this organism among host animal populations in the Canadian Arctic have generated concern regarding a potential increase in human infections. However, whole genome sequencing (WGS) of the organism was unable to identify a zoonotic origin for this case.

**Conclusions:**

Consideration should be given to *E. rhusiopathiae* as a cause of joint infections if the appropriate epidemiologic and host risk factors exist. Expanded use of WGS in other potential animal hosts and environmental sources may provide important epidemiologic information in determining the source of human infections.

## Background

*Erysipelothrix rhusiopathiae* is the causative organism of erysipeloid, a localized cutaneous infection [1–3]. Less commonly, it is known to cause systemic infection with or without endocarditis [1, 2, 4]. Infection is usually associated with occupational exposure to infected host animals and is most frequently reported following contact with domestic swine [1–3, 5]. We report a case of *E. rhusiopathiae* prosthetic joint infection (PJI) in a woman with exposure to multiple wild host species in the Canadian Arctic. *E. rhusiopathiae* has recently been linked to multiple muskox die-offs [6, 7] as well as the emergence of a new disease syndrome in Arctic fox [8] in these Arctic areas. This has generated concerns that an increase in human infections might be observed, especially in regions where exposure to potentially infected animals is expected to be greater [6].

### Case presentation

A 69-year-old woman was seen in follow-up at an outpatient orthopaedic clinic approximately 10 weeks after completing a 3-month course of antibiotic therapy for a right knee PJI due to *Brucella suis*, as reported previously [9]. She completely recovered following treatment of her *B. suis* PJI, but noted onset of acutely worsening right knee pain, warmth, and overlying redness 1 day prior to her scheduled follow-up. She was afebrile and systemically well.

An original total arthroplasty of the right knee was performed 12 years prior and she was diagnosed with a *B. suis* PJI after multiple synovial fluid aspirates grew the organism in 2015. She underwent irrigation and debridement with removal of all prosthetic components and implantation of a gentamicin, vancomycin, and ceftazidime impregnated static cement spacer. She completed 10 days of intravenous aminoglycoside therapy (initially tobramycin and then gentamicin) combined with oral doxycycline and rifampin. Oral antibiotics were continued for a total of 12 weeks. On initial follow-up, she had improved knee pain, no fever, normalization of inflammatory markers and a healed surgical wound.

The patient’s past medical history was otherwise significant for obesity, hypertension, gastroesophageal reflux disease and osteoarthritis with chronic back pain. Her medications were hydrochlorothiazide, ramipril, and pantoprazole. She had no known allergies. The patient lives on a remote island in the Canadian Arctic and works as an artist. She would regularly butcher wild meat (including caribou, muskox, seal and fish) and often consumed the meat and fish raw.

On physical examination, she was non-toxic and afebrile. Her right knee was swollen and erythematous. She had a static cement spacer at the time of this assessment and was not able to perform range of motion. There was no apparent drainage or visible sinus tracts on the right knee. The remainder of her physical examination was unremarkable.

Radiographs of the right knee revealed that the position of the intramedullary pins and large spacer was unchanged with no skeletal changes, however diffuse soft tissue swelling was evident. Erythrocyte sedimentation rate (ESR) and C-reactive protein (CRP) had risen from normal levels 3 months earlier to 49 mm/h and 171.4 mg/L respectively. Complete blood count did not show leukocytosis or neutrophilia. Serum creatinine was 66 μmol/L. A repeat *Brucella* IgG plus IgM standard agglutination assay performed in the previous month was negative with a titre of < 1:40. Given the clinical suspicion for recurrent PJI, a right knee arthrocentesis was performed in the orthopaedic outpatient clinic, which revealed a synovial fluid white blood cell count of 25,330 × 10^6^/L comprised of 87% polymorphonuclear cells. No organisms were seen on direct gram stain of the fluid.

A gram-positive bacillus was reported to be growing from liquid media culture 2 days after collection. The patient was taken back to the operating room the following day where she had irrigation and debridement of the right knee with removal of the previous prosthetic components and reinsertion of a static cement spacer with 3.6 g of tobramycin per bag of polymethylmethacrylate bone cement. Three bags of cement were used. No preoperative antibiotics were given. Once intra-operative tissue specimens had been collected, 2 g of cefazolin IV were given.

**Fig. 1:**
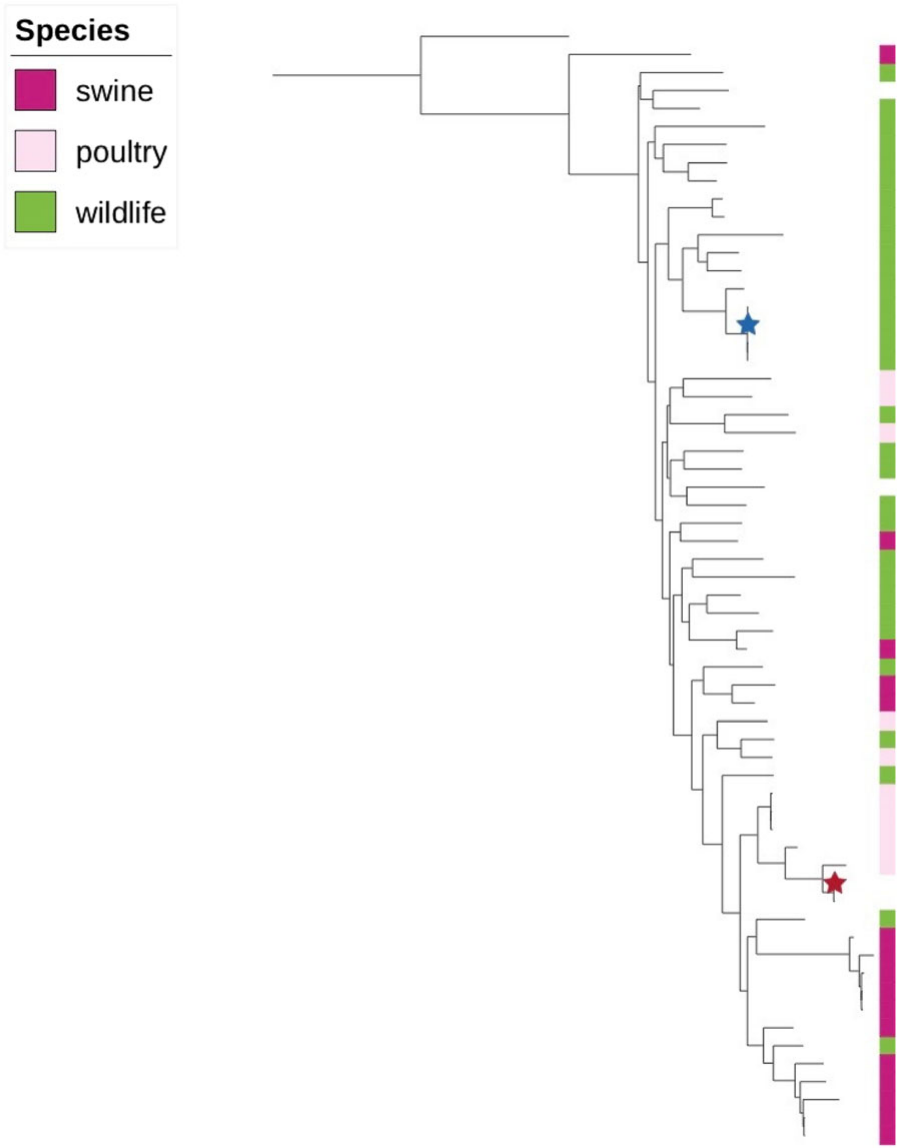
Whole genome sequencing comparison of the *Erysipelothrix rhusiopathiae* isolates from the present human prosthetic joint infection case (shown by red star) with other Clade 3 isolates from swine, poultry and various wildlife species. The tree is rooted to the reference Fujisawa strain (intermediate clade). The blue star highlights the strain found in multiple hosts from Banks, Victoria, and Prince Patrick Islands in Nunavut

The organism recovered from the pre-operative knee aspirate was confirmed as *E. rhusiopathiae* by 16S rRNA sequencing at the Provincial Laboratory for Public Health in Edmonton, Canada*.* This organism was also seen on direct gram stain and eventually isolated from all 5 intraoperative tissue specimens. Whole genome sequencing was performed on two of these isolates using the Illumina MiSeq platform. The 250 bp paired-end reads were assembled de novo using SPAdes (v3.10.1), and the assemblies compared to sequence data from previously isolated *E. rhusiopathiae* from various mammal carcasses from the Canadian Arctic, isolates from domestic swine and poultry as well as wild birds, mammals, and fish originating from various locations in North America and Europe [8]. This was done by generating a core genome alignment and phylogenetic tree using parsnp (Harvest Tools v1.2) (Fig. 1).

The isolate tested susceptible in vitro by E-test methods to ampicillin (minimum inhibitory concentration (MIC) 0.094 μg/mL), ciprofloxacin (MIC 0.047 μg/mL), and erythromycin (MIC 0.032 μg/mL) (interpreted according to the current M45 Clinical and Laboratory Standards Institute clinical breakpoints) and was reported resistant to vancomycin as this is an intrinsic characteristic. Four blood cultures, which were collected on the same day of this visit to the orthopedic outpatient clinic and prior to the administration of antibiotics, remained negative after 5 days of incubation. An echocardiogram was not performed as it was felt that the patient was unlikely to have endocarditis. She was initially treated with intravenous ceftriaxone 1 g daily but was switched to intravenous penicillin G once susceptibility results were available. She completed 6 weeks of intravenous antibiotic therapy and subsequently completed an additional 6 weeks of oral amoxicillin 1 g three times daily.

On the last day of her oral antibiotic therapy she was reviewed as an outpatient and was clinically well with no fever and resolution of knee pain. Her surgical wound had healed well with no evidence of recurrent infection on physical examination. Her CRP and ESR had normalized to 1.4 mg/L and 13 mm/h respectively. She was again seen in follow-up 4 months later (approximately 8 months after her last surgery) and remained well with no clinical evidence of relapse and CRP and ESR remaining within normal limits at 0.9 mg/L and 15 mm/h respectively. The patient declined a second stage revision procedure and remained clinically well 1 year after her last surgery.

## Discussion

*E. rhusiopathiae* is a ubiquitous, non-spore forming, facultative intracellular, gram-positive bacillus with a global distribution [2]. While *E. rhusiopathiae* is a commensal organism and a pathogen of a variety of domestic and wild animals, it is also known to exist as an environmental saprophyte [1]. Human infection with this organism is considered a zoonosis [1, 2]. The clinical spectrum of *E. rhusiopathiae* infection in humans consists of three major forms of disease: localized cutaneous infection, diffuse cutaneous infection, and systemic infection. Localized cutaneous infection, also known as erysipeloid, is the most common presentation and is usually described as a subacute cellulitis at the site of inoculation, which often resolves spontaneously within 3 weeks [1, 3]. Diffuse cutaneous disease and systemic disease that involves bacteremia with or without endocarditis is uncommon [4]. Other uncommon manifestations reported include pneumonia, abscesses, meningitis, endophthalmitis, osteomyelitis, and septic arthritis [1, 2].

Joint infection is a common occurrence in animals infected with *E. rhusiopathiae* [5]*.* However, septic arthritis of both native and prosthetic joints is a rarely reported manifestation of the disease in humans. Only 6 native joint infections and 4 PJIs with this organism have been reported previously, which are summarized in Table 1. The majority of clinical presentations were of a large joint chronic monoarthritis that developed over several months. Two cases had an acute presentation within days of presumed exposure, one after a penetrating injury to the shoulder [10] and the other occurring early after arthroscopic knee surgery [11]. Systemic symptoms and signs were absent in most cases, with only 2 patients reporting fever. CRP was measured in most and was moderately elevated.

**Table 1:** Summary of previously reported *E. rhusiopathiae* joint infections in the literature

Ref.	Age	Sex	Comorbid conditions	Joint	Possible Exposure	Presumed Mechanism of Joint Infection	Fever	CRP mg/L	X-Ray Findings	Antibiotic Therapy	Surgical Intervention	Follow-up period	Outcome
[10]	55	M	CRF, hemodialysis	Native shoulder	Work as butcher	Contiguous spread	NR	NR	NR	IV penicillin G × 3 weeks	Arthrotomy and irrigation	Unknown	No relapse
[27]	67	M	DM, CLL	Native elbow	Unknown	Unknown	No	NR	NR	None	Arthroscopic irrigation	Unknown	No relapse
[11]	18	M	None	Native knee	Knee laceration on seashore rock 2 months prior	Contiguous spread	No	146.7	NR	IV penicillin G + IV ciprofloxacin × 5 weeks then PO clindamycin + PO ciprofloxacin × 16 weeks	Arthroscopic irrigation and debridement	5 months	No relapse
[28]	41	F	Systemic and intra-articular steroid use for SLE	Native knee	Cleaning Koi fish pond weekly in prior 18 months	Contiguous spread	No	30.6	NR	IV penicillin G × 4 weeks then PO ciprofloxacin × 2 weeks	Arthrotomy and synovectomy	12 months	No relapse
[19]	76	M	Previous Aortic valve replacement	Native knee	Unknown	Hematogenous seeding	No	NR	NR	IV penicillin G × 4 weeks	None	Unknown	No relapse
[29]	5	M	None	Native hip	Unknown	Unknown	No	24	Increase in medial joint space	PO amoxicillin-clavulanate × 21 days	Arthrotomy and irrigation	4 months	No relapse
[30]	76	M	Long term high dose steroid use for RA and lupus nephritis	Prosthetic knee	Prior work with pig, cow, kangaroo, and penguin hides	Unknown	NR	27	Prosthetic misalignment	IV penicillin G + IV levofloxacin × 3 weeks then PO clindamycin + PO levofloxacin × 7 weeks	2 stage exchange with 2 chains of 30 gentamicin beads in tibial cavity and gentamicin spacer	12 months	No relapse
[31]	73	F	Unknown	Prosthetic hip	Owned a hunting dog	Unknown	NR	17.4	Radiolucency between femoral cement mantle and bone	IV penicillin G + IV × 3 weeks then PO amoxicillin × 8 weeks	2-stage exchange with gentamicin + clindamycin spacer	4 months	No relapse
[12]	68	F	Alcoholism, systemic steroid use	Prosthetic knee	Feeding swine	Contiguous spread	No	74	Advanced osteolysis of tibia, femur and patella	IV imipenem + IV ofloxacin × 2 weeks then PO clindamycin + PO ofloxacin × 24 weeks	Prosthesis removal with gentamicin + vancomycin spacer and eventual definitive arthrodesis	32 months	No relapse Arthrodesis
[32]	82	M	Unknown	Prosthetic knee	Unknown	Unknown	No	NR	Prosthesis loosening	IV ceftriaxone × 3 months for initial episode IV ceftriaxone + PO levofloxacin × 12 weeks for recurrrence	1-stage exchange for initial episode 2-stage exchange for recurrence	2 years	Relapse 1 month after initial antibiotic course. No relapse after 2-stage exchange

*Abbreviations: CRF* chronic renal failure, *DM* Diabetes mellitus, *CLL* chronic lymphocytic leukemia, *IV* intravenous, *NR* not reported, *PO* oral, *RA* rheumatoid arthritis, *Ref*. reference

A previously surmised finding is that many of the reported cases had immunosuppressive conditions including chronic lymphocytic leukemia, systemic lupus erythematosus, diabetes mellitus, chronic renal failure, hemodialysis, and corticosteroid use that likely predisposed them to invasive *E. rhusiopathiae* infection [12]. In this case, no immunosuppressive conditions were known to exist. However, previous and recent surgery as well as the presence of prosthetic material also likely increase patient propensity to develop intra-articular infection with this organism, if conditions for exposure exist.

A definitive diagnosis of *E. rhusiopathiae* infection largely still relies on culture techniques, which have variable sensitivity depending on the organism burden present in the specimen collected [1]. While organisms were only seen on direct gram stain of surgical tissues from the current joint infection case and specimens of one of the previously reported cases, *E. rhusiopathiae* was eventually recovered from all cases by culture methods. Identification using basic and automated biochemical systems as well as mass spectrometry technology, such as matrix assisted laser desorption/ionization time of flight, reliably identifies this organism [13]. Molecular diagnostic techniques, including sequencing of the conserved regions of the gene encoding 16S rRNA, are also a specific way to confirm identification [14]. Use of species-specific molecular diagnostic tests (i.e. polymerase chain reaction) directly on clinical specimens such as joint fluid may also be a sensitive and specific way to detect the presence of *E. rhusiopathiae* genetic material, particularly if there is high clinical suspicion and culture methods fail to recover the organism.

*E. rhusiopathiae* is intrinsically resistant to aminoglycosides and vancomycin but susceptible to penicillins, broad spectrum cephalosporins and fluoroquinolones, with no documented resistance to these agents [1, 2, 15]. The treatment of choice for localized and systemic infections is penicillin or ampicillin [1–4]. The ideal antibiotic regimen and duration for joint infections with this organism is unknown. It is recommended that native joint infections caused by other more common organisms be managed with a combination of drainage or surgery and antibiotic therapy [16, 17], and that PJIs be managed with surgery and antibiotics [18]. Management of previously reported *E. rhusiopathiae* joint infection cases has largely involved a combination of surgical intervention and antibiotic therapy (Table 1). Antibiotic decisions should be based on in vitro susceptibility results and individualized for each patient.

Transmission of *E. rhusiopathiae* to humans occurs after exposure to animals carrying or infected with the organism, or to their products [1–4]. Transmission following exposure to environmental sources harbouring the organism has also been reported [1]. The organism usually enters through non-intact skin, although infection following ingestion has been reported as well [1, 3]. In 4 of the 10 previously reported joint infection cases, *E. rhusiopathiae* was thought to gain access to the affected joint by contiguous spread from an overlying skin infection. One joint was presumed to be have been seeded hematogenously from an infected aortic valve [19]. The pathogenesis of the remaining 4 cases was unknown. The pathogenesis of the present case is unclear, however she did have a healing operative wound from the recent surgery when she reported handling wild muskox and other animal meat. *E. rhusiopathiae* may have been inoculated into this wound while she was handling meat from these potential source animals and, like previous cases, spread contiguously into her knee joint.

Most infections with *E. rhusiopathiae* are described in the setting of an occupational exposure to domestic animals with veterinarians, butchers, farmers, and fishermen being among those at highest risk [1, 2, 20]. Swine are described as the major reservoir for human infection that occurs in occupational settings, however multiple other animals are known to carry the organism, including Arctic marine mammals, fish, foxes and muskoxen [1, 2, 5, 6, 8]. Therefore, hunting and food practices of local populations in the Canadian Arctic are also known to increase risk for infection with *E. rhusiopathiae* [1, 6]. Multiple large-scale die-offs of muskoxen due to *E. rhusiopathiae* that have been reported in the Canadian Arctic in recent years, many on the same remote island as the case reported here [6], signal a changing epidemiology of this infection in mammals that may have an impact on human health in the region.

A comparison of whole genome sequences (WGS) generated from *E. rhusiopathiae* isolates found in these muskox carcasses between 2010 and 2013 previously revealed that a single strain was associated with die-off events across two large islands [21]. As part of an ongoing surveillance program in the Canadian arctic, further isolates collected in 2017 from muskoxen and seals on the same islands, and from muskoxen on a neighbouring but distant island identified that this same strain is still broadly circulating (Mavrot et al., unpublished). However, based on WGS comparisons, the isolate from this human case of PJI is not genetically related to the *E. rhusiopathiae* strain found in these arctic animal species and is more closely related to isolates found in poultry species as well as swine isolates from Canada and Belgium (Fig. 1), although the patient had no reported exposure to poultry or swine. This suggests that the patient acquired her infection from another animal or environmental source which WGS was not able to elucidate in this case given lacking epidemiologic linkages. Further investigations into the epidemiology of this pathogen in the Canadian Arctic, including the use of WGS and expanding testing to migratory birds and fish, could help to assess the likelihood of other animal and/or environmental sources as the cause of human zoonotic infections [21].

The prevalence of zoonotic infections in humans, like those caused by *E. rhusiopathiae,* is influenced by a number of factors including the burden of disease within the animal reservoir(s) [22, 23]. Animals may become more susceptible to a particular organism as changes to host-environment-pathogen interactions occur [6, 23]. One such driving factor cited for the changing epidemiology of many zoonotic infections is climate change [22–24]. It has been postulated that the prevalence of *E. rhusiopathiae* in certain animal host populations in the Canadian Arctic, such as muskoxen, is increasing partly as a result of climate change [6]. Kutz et al. who have been examining the epidemiology of *E. rhusiopathiae* in the Canadian Arctic expressed concern about the potential for zoonotic infection and emphasized the need for public health education and messaging to ensure that these important food sources continue to be harvested and handled in a way that prevents human exposure [6]. Guidelines for hunters in the Canadian North recommend that animals found dead not be touched or eaten, advise against cutting into animal parts that look abnormal, and whenever in doubt, to cook the meat or fish well [25, 26].

Infections with *E. rhusiopathiae* are not reportable to local or national health authorities in Canada. Without an epidemiological database for tracking infections, it is unknown if incidence of this infection in humans is increasing. Nonetheless, changes to the burden of disease in host animal populations are likely to result in an increased risk to those who rely on and are exposed to these animals and their environment on a regular basis. Our case experienced consecutive zoonotic infections of a prosthetic joint with *B. suis* followed by *E. rhusiopathiae*. Given the spread of *E. rhusiopathiae* among local wildlife in the Canadian Arctic, and the increased potential for human infection, consideration should be given to public health surveillance of this infection in this geographic region.

## Conclusion

*E. rhusiopathiae* joint infection is a rarely reported clinical entity, usually occurring in humans when host defences are debilitated. The present case occurred after exposure to the products of various potential host animals in the Canadian Arctic including muskoxen, which have suffered large-scale die-offs in recent years due to infection with this organism. Changing environmental conditions may be contributing to the increased burden of carriage or disease in certain host populations, thereby increasing the risk of infection to those who regularly interact with these animals or their environments. Since this infection is not a notifiable disease in Canada, consideration should be given to making it an infection under public health surveillance to allow the tracking of future human cases. Finally, expanding testing and subsequent WGS to other potential vertebrates and other environmental sources is likely to provide important epidemiologic information in determining the source of human infections.

## Abbreviations

CRP: C-reactive protein
ESR: Erythrocyte sedimentation rate
MIC: Minimum inhibitory concentration
PJI: Prosthetic joint infection
rRNA: Ribosomal ribonucleic acid
WGS: Whole genome sequencing
